# Metabolite Profiling Identifies Candidate Markers Reflecting the Clinical Adaptations Associated with Roux-en-Y Gastric Bypass Surgery

**DOI:** 10.1371/journal.pone.0007905

**Published:** 2009-11-19

**Authors:** David M. Mutch, Jens C. Fuhrmann, Dietrich Rein, Jan C. Wiemer, Jean-Luc Bouillot, Christine Poitou, Karine Clément

**Affiliations:** 1 Nutriomique U872 team 7, Institut National de la Santé et de la Recherche Médicale, Paris, France; 2 Centre de Recherche des Cordeliers, Université Pierre et Marie Curie – Paris 6, Paris, France; 3 metanomics GmbH, Berlin, Germany; 4 metanomics Health GmbH, Berlin, Germany; 5 Hôtel-Dieu Hospital Surgery Department, Assistance Publique-Hôpitaux de Paris, Paris, France; 6 Pitié-Salpêtrière Hospital Nutrition and Endocrinology Department, Assistance Publique-Hôpitaux de Paris, Paris, France; University of Las Palmas de Gran Canaria, Spain

## Abstract

**Background:**

Roux-en-Y gastric bypass (RYGB) surgery is associated with weight loss, improved insulin sensitivity and glucose homeostasis, and a reduction in co-morbidities such as diabetes and coronary heart disease. To generate further insight into the numerous metabolic adaptations associated with RYGB surgery, we profiled serum metabolites before and after gastric bypass surgery and integrated metabolite changes with clinical data.

**Methodology and Principal Findings:**

Serum metabolites were detected by gas and liquid chromatography-coupled mass spectrometry before, and 3 and 6 months after RYGB in morbidly obese female subjects (n = 14; BMI = 46.2±1.7). Subjects showed decreases in weight-related parameters and improvements in insulin sensitivity post surgery. The abundance of 48% (83 of 172) of the measured metabolites changed significantly within the first 3 months post RYGB (p<0.05), including sphingosines, unsaturated fatty acids, and branched chain amino acids. Dividing subjects into obese (n = 9) and obese/diabetic (n = 5) groups identified 8 metabolites that differed consistently at all time points and whose serum levels changed following RYGB: asparagine, lysophosphatidylcholine (C18:2), nervonic (C24:1) acid, p-Cresol sulfate, lactate, lycopene, glucose, and mannose. Changes in the aforementioned metabolites were integrated with clinical data for body mass index (BMI) and estimates for insulin resistance (HOMA-IR). Of these, nervonic acid was significantly and negatively correlated with HOMA-IR (p = 0.001, R = −0.55).

**Conclusions:**

Global metabolite profiling in morbidly obese subjects after RYGB has provided new information regarding the considerable metabolic alterations associated with this surgical procedure. Integrating clinical measurements with metabolomics data is capable of identifying markers that reflect the metabolic adaptations following RYGB.

## Introduction

Obesity is characterized by the accumulation of excess body fat to an extent that health is adversely affected via the development of co-morbidities. Due to a scarcity of validated and safe therapies, Roux-en-Y gastric bypass (RYGB) has become an increasingly effective treatment for severely obese patients [1], [2]. Following this procedure, patients experience a significant loss of weight; however, an unexpected and major outcome with gastric bypass surgery was that deaths related to coronary heart disease and diabetes were reduced by more than 50% and 90%, respectively [3]. Furthermore RYGB results in significant metabolic changes that lead to major improvements in blood glucose and insulin levels, insulin sensitivity and hormonal responses, as well as decreasing inflammatory markers [4], [5]. Lipid metabolism is also modified following the procedure, as demonstrated by decreases in total cholesterol and LDL-cholesterol, and increases in HDL-cholesterol [6]. Significant changes in the secretion of gastric and intestinal peptides such as glucagon-like peptide-1 (GLP-1), ghrelin, and peptide YY have also been demonstrated following RYGB [7], [8]. While a growing body of evidence supports the overall health benefits of bariatric surgery to treat morbid obesity, such a procedure is also associated with risks including mortality (<1%) and several complications such as venous thromboembolism, gallstone formation, and nutritional deficiencies [9]. Taken together, this procedure has a significant impact on a patient's whole-body metabolism and is therefore a unique model to understand the physiological and molecular mechanisms underlying the observed metabolic alterations.

Unraveling the immediate and long-term adaptations associated with gastric bypass surgery has proved challenging, predominantly because the consequences of this procedure include caloric restriction, diminished nutrient absorption, reduced adipose mass, modified gut hormone signaling, and changes in whole-body glucose metabolism that can each cause numerous physiological and metabolic adaptations. Because of a growing interest to treat type-II diabetes with gastric bypass surgery, the rapid and long-term improvement of insulin sensitivity and the reduction of diabetes in subjects post surgery has become a primary axis of interest. Several hypotheses have been postulated to explain the improved insulin sensitivity witnessed post surgery, and include the altered secretion of gut hormones [10], modifications in intestinal gluconeogenesis [11], and changes in intramyocellular lipid content [12]. While the definitive explanation for improved insulin sensitivity post RYGB remains unclear, it is most probably a combination of the aforementioned hypotheses.

One informative approach that has not yet been used to study the metabolic adaptations following RYGB is metabolite profiling, despite the fact that this approach has successfully generated new knowledge regarding the metabolic modifications associated with obesity and diet-induced weight loss [13]–[16]. Our present work has analyzed the metabolite profiles in the serum of morbidly obese female subjects who underwent RYGB. Subjects were further sub-divided into obese (OB) and obese/diabetics (OB/D) to identify metabolites associated with surrogates of steady-state insulin sensitivity. Our results provide new knowledge regarding the global alterations in serum metabolite profiles post RYGB and position nervonic acid as a possible marker of insulin sensitivity.

## Materials and Methods

### Ethics Statement

The Ethics Committees of the Hôtel-Dieu Hospital approved the clinical investigations and all subjects gave written consent.

### Subjects

Fourteen obese women (11 Caucasian, 2 Caribbean from French Antilles, and 1 African) involved in a gastric surgery program were prospectively recruited from the Ile-de-France region between 2005 and 2006 in the Department of Nutrition, Center of Reference for Medical and Surgical Care of Obesity, CREMO, Hôtel-Dieu (Paris, France). Patients met the criteria for obesity surgery, i.e. BMI≥40 kg/m^2^ or ≥35 kg/m^2^ with at least two co-morbidities (hypertension, type-II diabetes, dyslipidemia or obstructive sleep apnea syndrome). The preoperative evaluation included medical history, physical, nutritional, metabolic, cardiopulmonary, and psychological assessments. Subject weight was stable (i.e. variation of less than ±2 kg) for at least 3 months prior to operation. Subjects did not demonstrate evidence of acute or chronic inflammatory disease, infectious diseases, viral infection, cancer and/or known alcohol consumption (>20 g per day). According to the criteria of fasting glycemia over 7 mM or the use of an anti-diabetic drug, 5 subjects were also type 2 diabetics (4 Caucasians and 1 Caribbean from French Antilles). These five subjects (referred to hereon as the OB/D group) were treated with metformin and hypolipemic drugs (either fibrates or statins). Two subjects were additionally treated with insulin. The duration of diabetes in these 5 subjects was 7.4±1.0 years (range between 5–11 years). In the non-diabetic group (referred to hereon as the OB group), none were treated with hypolipemic drugs. Furthermore, an oral glucose tolerance test (OGTT) was systematically performed before RYGB and confirmed that all patients in the OB group had glucose levels less than 11 mmol/l (200 mg/dl) in the two hours following a 75 g oral glucose challenge.

### Clinical Assessment

All patients were assessed prior to Roux-en-Y surgery (i.e. baseline or T0) and at 3 months and 6 months post surgery (T3 and T6). Blood samples were obtained at each time point and stored at −20°C until used to assess lipid, insulin and glucose values (enabling the determination of insulin sensitivity parameters), leptin, and many other factors, outlined in [17]. Fat free body mass and adiposity were determined by DXA (DEXA, GE Lunar Prodigy Corporation, Madison, WI, USA) and resting energy expenditure (REE) by indirect calorimetry after 12 hours fasting (Deltatrac, Datex, France). The Homeostasis Model Assessment (HOMA) was used to estimate steady state beta cell function (HOMA%B) and insulin sensitivity (HOMA%S) [18]. HOMA insulin resistance (IR) was determined using the HOMA Calculator v2.2.2 (http://www.dtu.ox.ac.uk/). This index has been validated in different populations (including normal-weight, and obese type 2 diabetic and non-diabetic patients) in comparison with the euglycemic-hyperinsulinemic clamp values [19]. As such, the HOMA represents a useful index when studying morbidly obese individuals in whom the evaluation of insulin sensitivity using the clamp technique has understandable technical limitations due to extreme BMI.

### Dietary Assessment

Caloric intake and macronutrient proportions were evaluated by a registered dietician in the hospital nutrition department. Multivitamins and iron supplements were provided to avoid deficiencies, which are a well-known secondary effect of this bariatric surgery [20]. Blood measurements were performed before and after the surgery to adjust the dose of oral supplements. Serum iron, ferritin, the coefficient of saturation of iron in transferrin, vitamins (A, D, E, B1, B12, B9), micronutrients (selenium and zinc), and calcium were measured using routine bio-clinical tests. All patients were in the normal ranges for all aforementioned parameters at the different time points.

### Metabolite Analysis

Three types of mass spectrometry analyses were applied to all samples. GC-MS (gas chromatography-mass spectrometry; Agilent 6890 GC coupled to an Agilent 5973 MS-System, Agilent, Waldbronn, Germany) and LC-MS/MS (liquid chromatography-MS/MS; Agilent 1100 HPLC-System (Agilent, Waldbronn, Germany) coupled to an Applied Biosystems API4000 MS/MS-System (Applied Biosystems, Darmstadt, Germany)) were used for broad profiling, as described in [21]. SPE-LC-MS/MS (solid phase extraction-LC-MS/MS; Symbiosis Pharma (Spark, Emmen, Netherlands) coupled to an Applied Biosystems API4000 MS/MS-System (Applied Biosystems, Darmstadt, Germany)) was used for the determination of catecholamine and steroid levels. In this study, 242 metabolites fulfilled quality criteria for relative quantification, and absolute quantification was performed for an additional 14 metabolites. From a total of 256 metabolites, 172 were known metabolites, and 84 analytes were not chemically identified with sufficient certainty (i.e. thus considered in the present study to be unknown analytes). In this study, statistical analysis and interpretation focused on known metabolites only. To aid interpretation, metabolites were grouped into 11 biological categories: amino acid and related; carbohydrates and related; catecholamines and related; complex lipids and related; energy metabolism and organic acids; fatty acids (free and from lipids); purines, pyrimidines and related; steroids and related; vitamins, cofactors and related; miscellaneous; and unknown metabolites.

Technical reference samples were measured in parallel with the study samples in order to allow the relative quantification of metabolites in the study samples. These technical reference samples were generated by randomly pooling serum from 15 healthy female controls (average age = 48.9±2.1; BMI range of 21-24). A relative quantification for each metabolite was obtained by normalizing peak intensity in the study samples to the median peak intensity of the corresponding metabolite in the technical reference samples measured in the same batch.

### Metabolite Profiling by GC-MS and LC-MS/MS

Proteins were removed from serum samples (60 ul) by precipitation. Subsequently polar and non-polar fractions were separated for both GC-MS and LC-MS/MS analysis by adding water and a mixture of ethanol and dichloromethane. For GC-MS analyses, the non-polar fraction was treated with methanol under acidic conditions to yield the fatty acid methyl esters derived from both free fatty acids and hydrolyzed complex lipids. The polar and non-polar fractions were further derivatized with O-methyl-hydroxyamine hydrochloride (20 mg/ml in pyridine, 50 ul) to convert oxo-groups to O-methyloximes and subsequently with a silylating agent (MSTFA, 50 ul) before GC-MS analysis [22]. For LC-MS/MS analyses, both fractions were reconstituted in appropriate solvent mixtures. High performance LC (HPLC) was performed by gradient elution using methanol/water/formic acid on reversed phase separation columns. Mass spectrometric detection technology was applied as described in the patent US 7196323, which allows targeted and high sensitivity “Multiple Reaction Monitoring” profiling in parallel to a full screen analysis. Use of the term “Minor” in figure and table legends indicates that quantification can be affected by the co-occurrence of metabolites exhibiting identical characteristics in the analytical methods. Literature data and/or comparison with alternative methods (e.g. LC-MS/MS, GC-MS) suggest that such metabolites are present at minor levels only.

### Quantification by SPE-LC-MS/MS

Steroids and their related metabolites were measured by online SPE-LC-MS/MS. Catecholamines and their related metabolites were measured by online SPE-LC-MS/MS, as described by Yamada et al [23]. Quantification was performed using stable isotope-labeled standards.

### ANOVA and Data Correction

**Figure 1:**
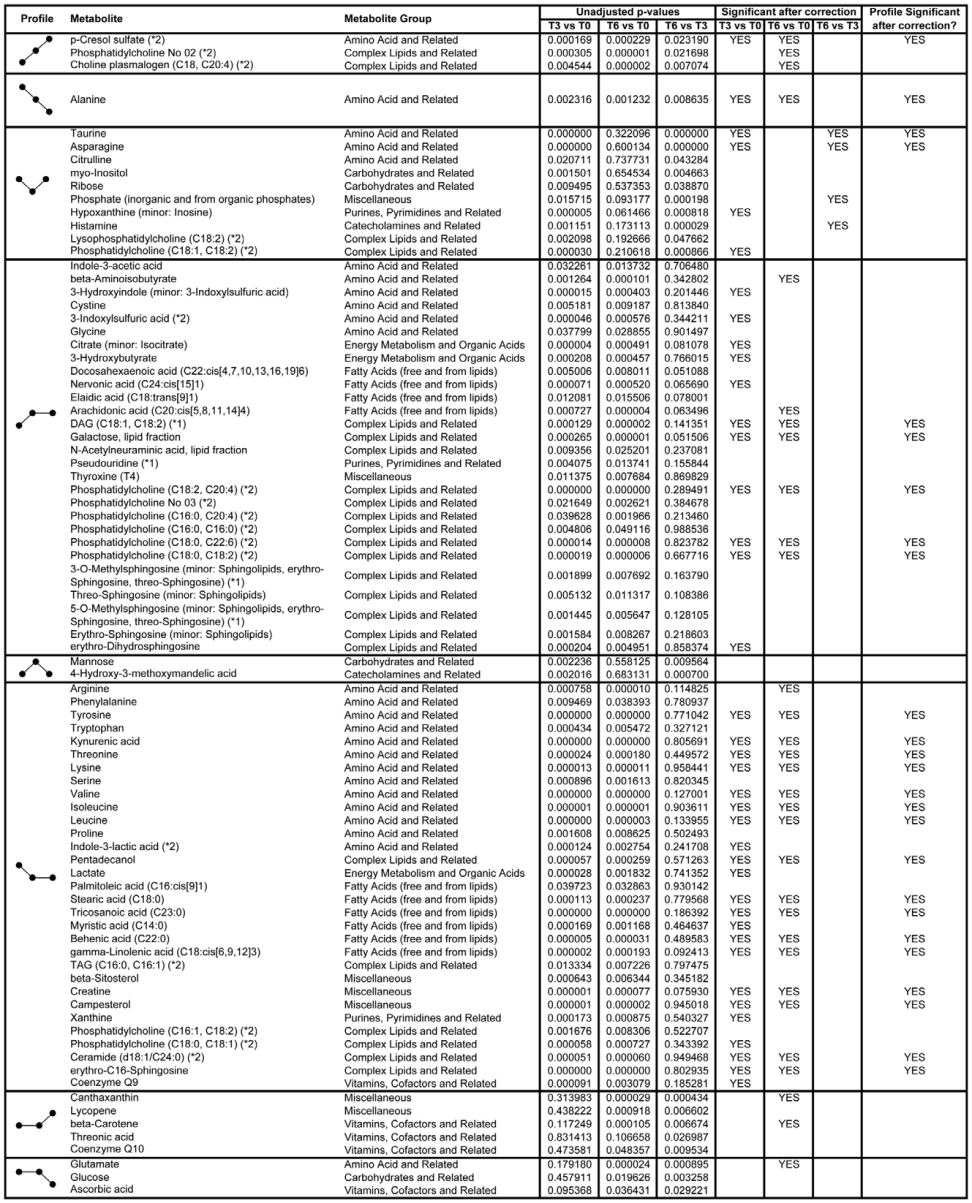
Various profiles for metabolites that change significantly at some point following RYGB. A profile is characterized by 3 dots, which represent T0 (prior to RYGB), T3 and T6 (post surgery). An angled slope between two time points indicates a significant change (p<0.05) and a flat slope between two time points indicates non-significant changes. Based on data derived from the mixed-effects ANOVA using all 14 subjects together. (*1): Structure annotation is based on strong analytical evidence (combinations of chromatography, mass spectrometry, chemical reactions, deuterium-labeling, database and literature search, as well as comparisons to similar/homologue/isomeric reference compounds). (*2): Metabolite exhibits identical qualitative analytical characteristics (chromatography and mass spectrometry) compared to status (*1). Further structural and analytical investigations of this metabolite - also in comparison to structurally identified or status (*1) metabolites - are still pending.

An ANOVA model was developed to determine differences in metabolite abundance over time (T0, T3, and T6) and between groups (OB vs. OB/D). Metabolite data was log_10_-transformed and a mixed-effects ANOVA was conducted for univariate data analysis. Two modeling approaches were applied, where both approaches began with fixed effects: sample_age (numeric, to account for serum storage time), group (categorical: OB or OB/D) and time (categorical: T0, T3, T6), and with random patient effect (categorical, patient 1–14). The second more general approach comprised an additional group:time interaction to account for possible subgroup-specific changes over time (i.e. RYGB having different effects on OB and OB/D subjects). The Akaike information criterion (AIC) was applied for metabolite-specific stepwise model evaluation and reduction of model complexity with regards to fixed effects (all fixed effects except time were considered optional). Experimental groups were compared and statistically evaluated by t-statistics of model contrasts. The estimated models were also used for data correction with regards to sample_age by metabolite-specific subtraction of linear sample_age estimations from log_10_-transformed data when required (i.e. when sample_age was not removed by AIC model complexity reduction). Analysis was conducted with statistical software R version 2.4.1 (Copyright (C) 2006 “The R Foundation for Statistical Computing”, http://www.r-project.org/). In **FIGURE 1**, unadjusted p-values are shown to indicate significance levels. Furthermore, a “YES” is indicated in the corresponding columns when changes remained significant (p<0.05) after Bonferroni correction for multiple testing.

### Multivariate Analysis of Metabolite Data

Partial least squares discriminant analysis (PLS-DA) of sample_age-corrected data for the 172 known metabolites was performed for classification analysis according to time (T0 vs. T3 vs. T6) and group (OB vs. OB/D, time-specific) using Umetrics SIMCA-P software (Version 11.0, Umetrics AB, Umea, Sweden). Model performance was quantified by Q2 values from leave-one-subject-out cross validation.

### Statistical Analysis of Clinical Data

The distribution of clinical data prior to surgery was tested using the Shapiro-Wilk W Test. Non-parametric statistical tests were used to assess clinical data over time (Mann-Whitney U-Test) and between groups (Friedman test); performed using GraphPad Prism version 4.0 software (GraphPad Software, Inc., California, USA). All data is presented as mean±SEM.

### Correlations between Metabolite and Clinical Data

Correlations between the abundance of 8 metabolites and two clinical parameters (BMI or HOMA-IR) were assessed with a Spearman correlation using JMP statistical software v5.1.2 (SAS Institute Inc., Cary, NC, USA).

## Results

### Clinical Characteristics of Subjects before and after RYGB

**Figure 2:**
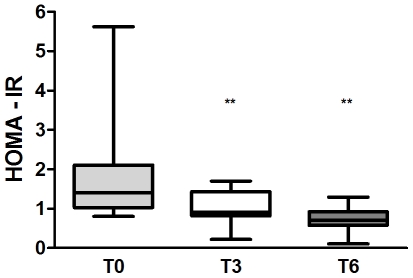
Estimates for HOMA-IR before and after RYGB. HOMA-IR was estimated for 12 subjects, 9 OB and 3 OB/D subjects. The OB/D subjects were treated with metformin and not with insulin. A significant reduction (T0 → T3, p = 0.014; T0 → T6, p = 0.001; T3 → T6, p = 0.123) in HOMA-IR occurred following RYGB, as assessed using a Friedman test. Prior to RYGB, significant variability in HOMA-IR estimations was observed between subjects (because OB and OB/D subjects are combined); however, post RYGB, all subjects demonstrated a major improvement in insulin sensitivity (illustrated by smaller error bars). Box plots indicate no outlying data (i.e. above or below the whiskers), and the band in the middle of the box indicates the median. ** *p*<0.01.

Our study population consisted of 9 obese (OB) subjects and 5 obese/diabetic (OB/D) female subjects, with an average BMI of 46.2±1.7. The OB/D subjects were all treated with metformin and 2 of these subjects were additionally treated with insulin. RYGB resulted in the expected and significant decreases in body mass index (BMI), body weight (kg), fat mass (kg), and fat free mass (kg) from T0 → T3 → T6 (**TABLE 1**). Resting energy expenditure (REE) was decreased in all subjects following RYGB. Additionally, circulating triglycerides and leptin levels were decreased post surgery. There was a small, but significant, decrease in HDL-cholesterol between T0 and T3; however, HDL-cholesterol levels recovered by T6; as further supported by a lack of change in Apo-A1 levels between T0 and T6. Total caloric intake was significantly decreased following RYGB; however, the relative proportion of protein, lipid and carbohydrate consumed in the diet remained stable post surgery. Since insulin injections prevent an accurate measure of endogenous insulin and glucose levels, the 2 OB/D subjects treated with insulin were removed prior to evaluating insulin resistance with the HOMA. In the remaining 12 subjects, circulating glucose and insulin levels decreased significantly post RYGB. A significant decrease in insulin resistance (evaluated by HOMA-IR) was found at T3 and this decrease continued (albeit less dramatically) between T3 and T6 (**FIGURE 2**). Changes in HOMA-IR corresponded with the expected increase in insulin sensitivity (HOMA%S) and a decrease in beta-cell function (HOMA%B). These improvements were observed in both OB and OB/D subgroups when considered independently (data not shown).

**Table 1:** Clinical data in subjects at all 3 time points (T0, T3, and T6) examined.

Parameter	T0	T3	T6
***n = 14 (all OB and OB/D subjects)***			
**Age (yrs)**	45.4±3.6		
**BMI (kg/m^2^)**	46.2±1.7	38.7±1.6^**^	35.1±1.7^ **^
**Weight (kg)**	125.4±4.2	104.6±3.9^ **^	95.1±4.0^ **^
**Fat mass (kg)**	59.3±3.5	46.9±3.2^ **^	39.2±2.9^ **^
**Free fat mass - FFM (kg)**	60.2±1.5	54.1±1.7^ **^	51.9±1.8^**^
**Resting Energy Expenditure (kcal/d/kg FFM)**	34.3±0.4	33.6±0.4^**^	32.8±0.3^**^
**Total Cholesterol (mmol/l)**	4.7±0.3	4.3±0.3	4.4±0.3
**HDL-Cholesterol (mmol/l)**	1.5±0.1	1.3±0.1^ **^	1.5±0.1
**Triglycerides (mmol/l)**	1.3±0.2	1.1±0.1^**^	1.0±0.1^**^
**Apolipoprotein A-1 (g/l)**	1.4±0.1	n/a	1.4±0.1
**Leptin (ng/ml)**	69.1±6.5	37.9±5.2^**^	24.2±4.0^**^
**Total Caloric Intake (kcal/d)**	1861±149.2	991±101.6^**^	1106±98.4^**^
**% Protein**	19.2±1.1	19.6±0.8	19.1±1.1
**Nutrient Intake % Lipid**	33.1±2.0	33.7±1.9	32.0±1.8
**% Carbohydrate**	47.6±2.2	46.7±1.9	47.5±2.8
***n = 12 (the two insulin treated OB/D subjects removed)***			
**Glucose (mmol/l)**	5.44±0.34	4.92±0.17^*^	4.53±0.10^**^
**Insulin (IU/l)**	15.6±3.5	7.8±1.0^**^	5.8±0.7^**^
**HOMA-IR**	2.0±0.4	1.0±0.1^**^	0.7±0.1^**^
**HOMA%S**	70.3±10.0	129.8±29.7^**^	207.8±66.2^**^
**HOMA%B**	134.5±16.9	101.0±12.1^**^	92.5±7.6^**^

All 14 subjects are included in the analysis of parameters related to body weight and lipids. Decreases after RYGB were observed for BMI, weight, fat mass, fat free mass, resting energy expenditure and triglycerides. Leptin was also decreased significantly. While HDL-cholesterol decreased from T0 to T3, HDL-Cholesterol levels recovered by T6 and are confirmed by the lack of change in Apo-A1 levels. Total caloric intake decreased after RYGB; however, the relative proportion of lipid, carbohydrate, and protein consumed remained stable. When considering the 12 subjects not treated with insulin, glucose and insulin levels decreased post RYGB. Estimates for HOMA-IR and HOMA%B decreased while HOMA%S increased after surgery. Data presented as mean±standard error. * represents p<0.1 and ** represents p<0.05, assessed by a Friedman test.

Subjects in the OB/D group showed further improvements in relation to their diabetic status. Prior to RYGB, HbA1c levels were 8.1%±0.3 and at T3 the HbA1c levels dropped to 6.8%±0.2 (p = 0.06). Blood glucose levels in three of the five subjects normalized after RYGB and anti-diabetic treatments were stopped, indicating a resolution of diabetes. The two OB/D subjects treated with insulin prior to surgery showed improvements post surgery (HbA1c levels dropped from 9.4% and 9.0% at T0 to 7.1% and 7.8% at T3); however insulin therapy was maintained and diabetes was not resolved.

### Global Metabolite Analysis

**Figure 3:**
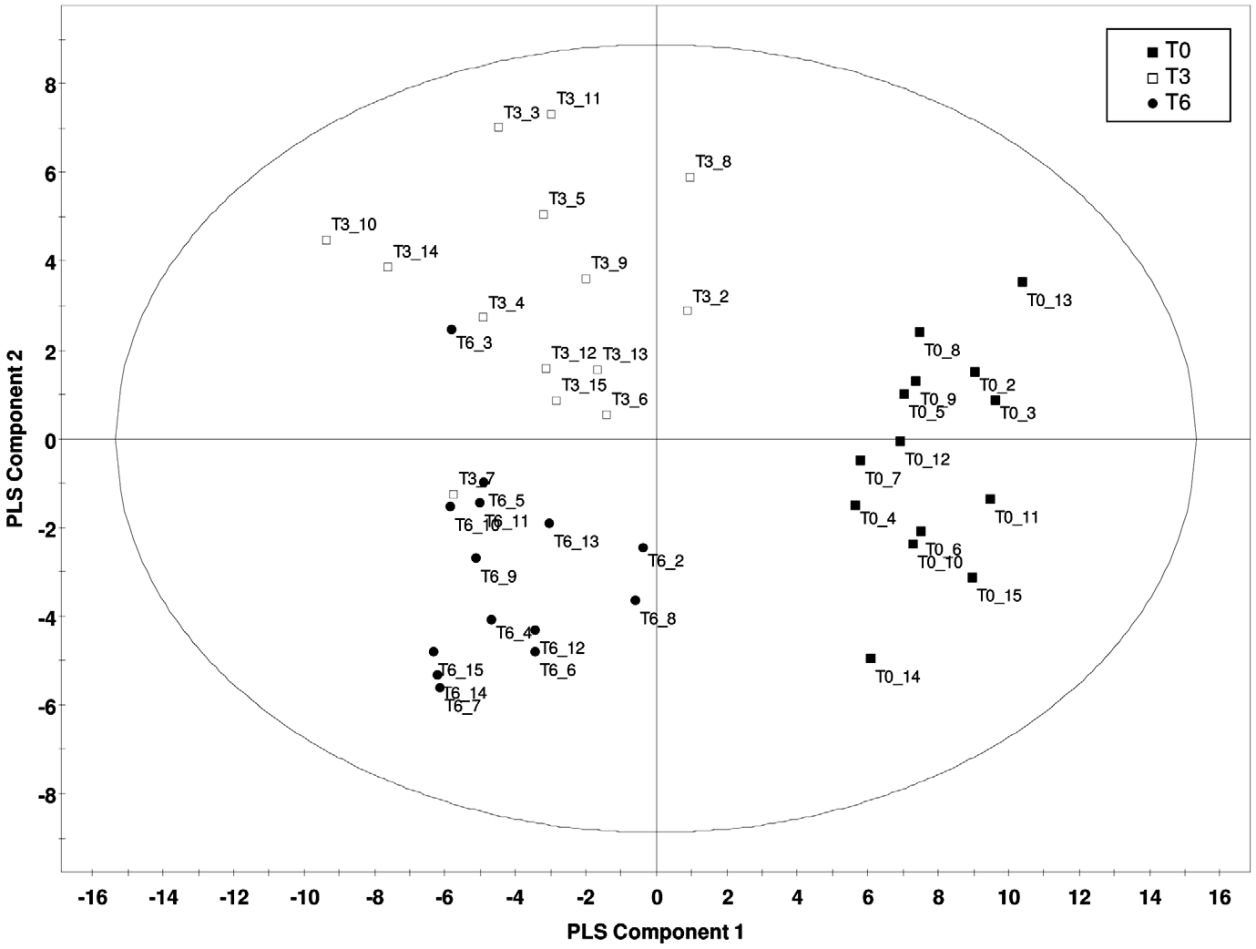
Partial least squares discriminant analysis of 172 known metabolites in 14 subjects distinguishing the three classes: pre-operative (T0) vs. 3 months after RYGB (T3) vs. 6 months after RYGB (T6). The first component distinctly classifies T0 vs. after RYGB (Q2 = 0.423), the second component further distinguishes T3 and T6 (Q2(cum) = 0.640 for two-component PLS-DA model). Model performance was quantified by Q2 values from leave-one-subject-out cross validation. Q2 depicts the fraction of the total variation that is predicted by each PLS component, while Q2(cum) depicts the cumulated fraction of the total variation predicted by the model.

Partial least squares discriminant (PLS-DA) analysis of the 172 known metabolites measured in the serum of 14 subjects led to a clear separation between the metabolite profiles before (T0), 3 months after (T3), and 6 months after (T6) gastric bypass surgery (similarly observed with principal component analysis – data not shown). The model, consisting of four components, had a R2X(cumulative) of 0.430 and a Q2(cumulative) of 0.806 (selected by leave-one-subject-out cross validation according to maximal Q2(cum)). **FIGURE 3** shows the first two out of four components (for first two components, R2X(cum) = 0.313, Q2(cum) = 0.640). Furthermore, there was a distinct separation between T0 and T3/T6 (first PLS-DA component with large Q2_comp1 = 0.423) and a further separation between the metabolite profiles at T3 and T6 with minor overlap in the second PLS-DA component. **FIGURE 1** lists the 83 metabolites that changed significantly at some point following RYGB in the 14 patients (mixed-effects ANOVA without group:time-interaction). 89 known metabolites did not change significantly following surgery. It is interesting to note that the serum abundance of 93% of these metabolites (77 of 83) changed significantly within the first 3 months post bypass surgery and that 77% of these metabolites (59 of 77) did not subsequently change between 3 and 6 months post surgery, despite the continued significant decreases in BMI and body fat mass. Amino acids and lipids were the two metabolite classes predominantly altered following RYGB, where amino acids were decreased and specific lipids were increased or decreased. Specifically, the branched-chain amino acids (leucine, isoleucine, and valine) decreased following RYGB. Further evidence for the regulation of valine catabolism in particular was provided by the increased serum abundance for β-aminoisobutyric acid, a product of valine catabolism (**FIGURE 1**). With regards to lipid metabolites, we observed increases in specific sphingosines, unsaturated fatty acids, and phospholipids, while ceramide (d18:1, C24:0), triglycerides, and saturated fatty acids decreased following RYGB.

### Obese versus Obese/Diabetic Subjects

**Figure 4:**
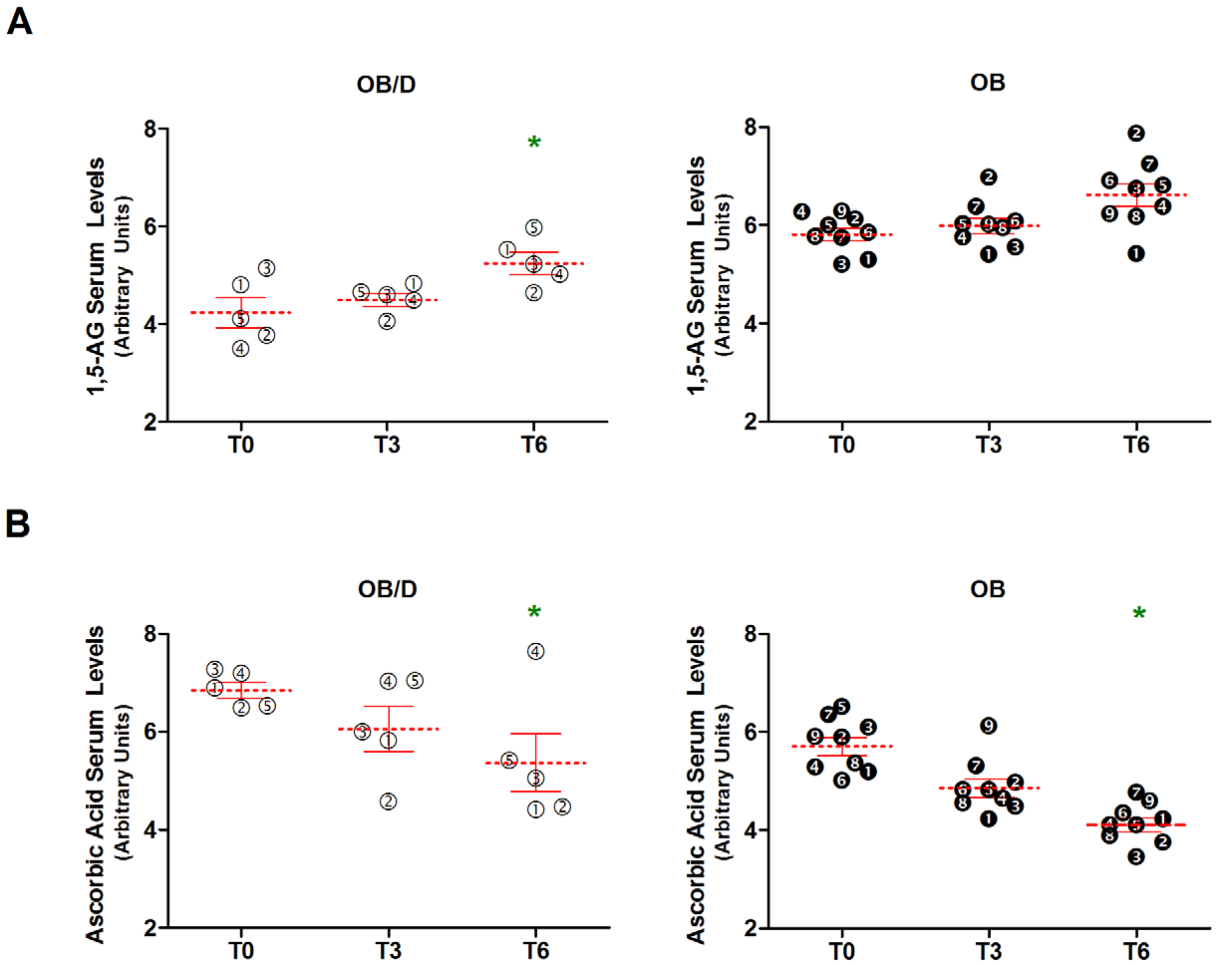
Increases in 1,5-anhydrosorbitol and decreases in ascorbic acid following RYGB. The top two metabolites distinguishing OB and OB/D subjects were identified with the mixed-effects ANOVA allowing for group:time-interactions, where * indicates *p*<0.05 (versus T0). White circles and black circles correspond to OB/D or OB subjects, respectively. Furthermore, symbols and numbers are consistently used for the same subject in Figures 4 & 5. Mean metabolite abundance±SEM is indicated in red. A) A significant increase in 1,5-anhydrosorbitol (1,5-AG) occurred at T6 vs. T0 in OB/D subjects, while a non-significant increase was seen in OB subjects (group:time interaction was allowed for by the linear model). B) For ascorbic acid, the ANOVA model did not detect group-specific changes over time (no group:time interaction), but indicated significant decreases from T0 to T6 in both groups. Overall, 1,5-anhydrosorbitol and ascorbic acid were significantly different at each time point between OB and OB/D subgroups (p<0.01, see **TABLE 2**).

As previously mentioned, the population consisted of 9 OB and 5 OB/D subjects; therefore, we subsequently compared the metabolite profiles of these two subgroups. PLS-DA displayed a separation between OB and OB/D groups at each time point before and after surgery (1 component PLS-DA model selected by cross validation for T0 and T3, 2 component model for T6; T0 → R2X = 0.156 and Q2 = 0.528; T3 → R2X = 0.146 and Q2 = 0.529; and T6 → R2X = 0.149 and Q2_comp1 = 0.585, Q2(cum) = 0.711). While many metabolites may contribute to this separation, we considered only those metabolites that differed between OB and OB/D subjects with p<0.05 at each time point according to the mixed-effects ANOVA, allowing group:time-interactions when statistically adequate. If the differences in metabolite abundance between OB and OB/D groups did not vary significantly with time (i.e. group:time interaction excluded by AIC), the same p-value appears at all three time points. We identified 33, 32, and 28 metabolites that differed significantly (p<0.05) between OB and OB/D subjects at T0, T3, and T6, respectively (**TABLE 2**). This is substantially more metabolites than expected by chance, where one could expect 9 false-positives per OB vs. OB/D comparison due to multiple testing at a 5% significance level. The difference between the 33, 32 and 28 significant changes in the dataset and 9 expected false-positives was found to be statistically significant (p = 3^e-11^ for T0, 1^e-10^ for T3 and 4^e-08^ for T6, binomial test). Seventeen metabolites were identified across all 3 lists. The top two metabolites common to each of the 3 lists were 1,5-anhydrosorbitol and ascorbic acid. Interestingly, 1,5-anhydrosorbitol is a biomarker currently used for assessing glycemic control. As blood glucose levels increase above the renal threshold for glucosuria, 1,5-anhydrosorbitol rapidly decreases [24]. The current dataset shows that 1,5-anhydrosorbitol levels are significantly lower in OB/D versus OB subjects (**FIGURE 4A**). Following RYGB, the levels of 1,5-anhydrosorbitol increased from T0→T3→T6, while glucose levels decreased. Furthermore, glucose also appears in **TABLE 2**, which serves to reinforce this result. The mixed-effects ANOVA model revealed a significant group:time interaction for 1,5-anhydrosorbitol. The second metabolite is ascorbic acid, whose abundance in serum decreased post surgery. OB/D subjects have greater levels of ascorbic acid versus OB subjects at all time points, despite both groups of subjects experiencing similar decreases in ascorbic acid levels post surgery (**FIGURE 4B**). No significant interaction between group:time was identified for ascorbic acid. Deficiencies in vitamins are a documented side-effect of gastric bypass surgery [25], [26], therefore all subjects consumed a daily multi-vitamin containing approximately 120 mg of vitamin C post surgery. Thus serum vitamin C differences between OB and OB/D groups in this study are most likely not related to differences in nutritional supplementation.

**Table 2:** Metabolite lists differentiating OB from OB/D subjects at each time point.

Prior to surgery (T0)			3 months post surgery (T3)			6 months post surgery (T6)		
Metabolite	p-value	Greater in	Metabolite	p-value	Greater in	Metabolite	p-value	Greater in
**1,5-Anhydrosorbitol**	0.00004	OB	**1,5-Anhydrosorbitol**	0.0002	OB	Valine	0.0004	OB/D
**Ascorbic acid**	0.0020	OB/D	**Ascorbic acid**	0.0020	OB/D	**Ascorbic acid**	0.0020	OB/D
Testosterone	0.0024	OB	11-Deoxycortisol	0.0032	OB/D	Leucine	0.0030	OB/D
Normetanephrine	0.0040	OB	Hypoxanthine (minor: Inosine)	0.0041	OB/D	**1,5-Anhydrosorbitol**	0.0032	OB
**Lycopene**	0.0043	OB	**Lycopene**	0.0043	OB	myo-Inositol-2-phosphate (minor: Fructose-6-phosphate, Glucose-6-phosphate, myo-Inositol-1-phosphate, myo-Inositol-4-phosphate)	0.0033	OB/D
**Asparagine**	0.0046	OB/D	**Asparagine**	0.0046	OB/D	**Lycopene**	0.0043	OB
**Lysophosphatidylcholine (C18:2)** (*2)	0.0059	OB	**Lysophosphatidylcholine (C18:2)** (*2)	0.0059	OB	**Asparagine**	0.0046	OB/D
Androstenedione	0.0062	OB	Phosphatidylcholine (C18:0, C18:1) (*2)	0.0064	OB/D	**Lysophosphatidylcholine (C18:2)** (*2)	0.0059	OB
Linoleic acid (C18:cis[9], [12]2)	0.0070	OB	**Cholic acid**	0.0088	OB/D	Proline	0.0061	OB/D
Phosphatidylcholine No 02 (*2)	0.0083	OB/D	**Cysteine (minor: Cystine)**	0.0091	OB/D	Xanthine	0.0082	OB/D
**Cysteine (minor: Cystine)**	0.0091	OB/D	**Phosphatidylcholine (C16:0, C18:2)** (*2)	0.0091	OB/D	**Cysteine (minor: Cystine)**	0.0091	OB/D
**Phosphatidylcholine (C16:0, C18:2)** (*2)	0.0091	OB/D	Valine	0.0115	OB/D	**Phosphatidylcholine (C16:0, C18:2) **(*2)	0.0091	OB/D
5-Hydroxy-3-indoleacetic acid (5-HIAA)	0.0116	OB/D	**p-Cresol sulfate** (*2)	0.0133	OB/D	Phenylalanine	0.0112	OB/D
**p-Cresol sulfate** (*2)	0.0133	OB/D	**Lactate**	0.0140	OB/D	**p-Cresol sulfate** (*2)	0.0133	OB/D
Lactate**	0.0140	OB/D	Ribose	0.0148	OB/D	**Lactate**	0.0140	OB/D
Phosphatidylcholine (C18:2, C20:4) (*2)	0.0146	OB/D	**Glucose-1-phosphate (minor: Glucose)**	0.0167	OB/D	**Glucose-1-phosphate (minor: Glucose)**	0.0167	OB/D
**Glucose-1-phosphate (minor: Glucose)**	0.0167	OB/D	**Pantothenic acid**	0.0176	OB/D	**Pantothenic acid**	0.0176	OB/D
**Pantothenic acid**	0.0176	OB/D	Taurine	0.0181	OB/D	Isoleucine	0.0209	OB/D
3-O-Methylsphingosine (minor: Sphingolipids, erythro-Sphingosine, threo-Sphingosine) (*1)	0.0176	OB	myo-Inositol-2-phosphate (minor: Fructose-6-phosphate, Glucose-6-phosphate, myo-Inositol-1-phosphate, myo-Inositol-4-phosphate)	0.0195	OB/D	Phosphatidylcholine (C18:0, C18:1) (*2)	0.0223	OB/D
threo-Sphingosine (minor: Sphingolipids)	0.0232	OB	Citrulline	0.0241	OB/D	Ornithine (minor: Arginine, Citrulline)	0.0237	OB/D
Threitol	0.0241	OB/D	Xanthine	0.0264	OB/D	**Nervonic acid (C24:cis** [**15**] **1)**	0.0265	OB
erythro-Sphingosine (minor: Sphingolipids)	0.0243	OB	**Nervonic acid (C24:cis** [**15**] **1)**	0.0265	OB	**Fructose-6-phosphate (minor: Glucose-6-phosphate, myo-Inositol-1-phosphate, myo-Inositol-2-phosphate, myo-Inositol-4-phosphate)**	0.0296	OB/D
5-O-Methylsphingosine (minor: Sphingolipids, erythro-Sphingosine, threo-Sphingosine) (*1)	0.0249	OB	**Fructose-6-phosphate (minor: Glucose-6-phosphate, myo-Inositol-1-phosphate, myo-Inositol-2-phosphate, myo-Inositol-4-phosphate)**	0.0296	OB/D	Testosterone-17-sulfate (minor: Dehydroepiandrosterone sulfate) (*2)	0.0299	OB
N-Acetylneuraminic acid, lipid fraction	0.0256	OB	Testosterone-17-sulfate (minor: Dehydroepiandrosterone sulfate) (*2)	0.0299	OB	Taurine	0.0309	OB/D
**Nervonic acid (C24:cis** [**15**] **1)**	0.0265	OB	gamma-Linolenic acid (C18:cis[6], [9], [12]3)	0.0300	OB/D	**Cholic acid**	0.0325	OB/D
**Cholic acid**	0.0279	OB	Phosphatidylcholine (C18:1, C18:2) (*2)	0.0327	OB/D	**Glucose**	0.0376	OB/D
**Fructose-6-phosphate (minor: Glucose-6-phosphate, myo-Inositol-1-phosphate, myo-Inositol-2-phosphate, myo-Inositol-4-phosphate)**	0.0296	OB/D	Threitol	0.0366	OB/D	Citrulline	0.0439	OB/D
Testosterone-17-sulfate (minor: Dehydroepiandrosterone sulfate) (*2)	0.0299	OB	**Glucose**	0.0376	OB/D	**Mannose**	0.0468	OB/D
Phytosphingosine	0.0302	OB	Sucrose	0.0390	OB/D			
**Glucose**	0.0376	OB/D	Ornithine (minor: Arginine, Citrulline)	0.0409	OB/D			
**Mannose**	0.0468	OB/D	**Mannose**	0.0468	OB/D			
Arginine	0.0469	OB	Phosphatidylcholine (C16:0, C16:0) (*2)	0.0498	OB			
Sphingomyelin No 02 (*2)	0.0484	OB						

Metabolites were identified using a mixed-effects ANOVA: 33 metabolites at T0, 32 metabolites at T3, and 28 metabolites at T6 (p<0.05), with indication of whether serum levels are higher in obese (OB) or obese/diabetic (OB/D) subjects. Those metabolites present in all three lists are indicated in bold font. The profiles for metabolites whose abundance changed post surgery are found in **FIGURE 1**. (*1): Structure annotation is based on strong analytical evidence (combinations of chromatography, mass spectrometry, chemical reactions, deuterium-labeling, database and literature search, as well as comparisons to similar/homologue/isomeric reference compounds). (*2): Metabolite exhibits identical qualitative analytical characteristics (chromatography and mass spectrometry) compared to status (*1). Further structural and analytical investigations of this metabolite - also in comparison to structurally identified or status (*1) metabolites - are still pending.

The 15 remaining metabolites that differed between OB and OB/D subjects at all time points were asparagine, cysteine, lysophosphatidylcholine (C18:2), phosphatidylcholine (C16:0, C18:2), nervonic acid (C24:cis[15]1), cholic acid, pantothenic acid, lycopene, lactate, testosterone-17-sulfate, p-Cresol sulfate, fructose-6-phosphate, glucose-1-phosphate, glucose, and mannose. We then determined whether the serum abundance of the aforementioned metabolites changed significantly post surgery, with the idea that these metabolites may then serve as candidate markers for the improved clinical characteristics seen post surgery. Of the 15 aforementioned metabolites, the abundance of 8 metabolites changed significantly after surgery (i.e. are found in **FIGURE 1**): asparagine, lysophosphatidylcholine (C18:2), nervonic (C24:1) acid, p-Cresol sulfate, lactate, lycopene, glucose, and mannose. Lycopene, lysophosphatidylcholine, and nervonic acid serum levels were higher in OB subjects, while asparagine, p-Cresol sulfate, lactate, glucose and mannose were higher in OB/D subjects.

### Relationship between Metabolites and Clinical Data

**Figure 5:**
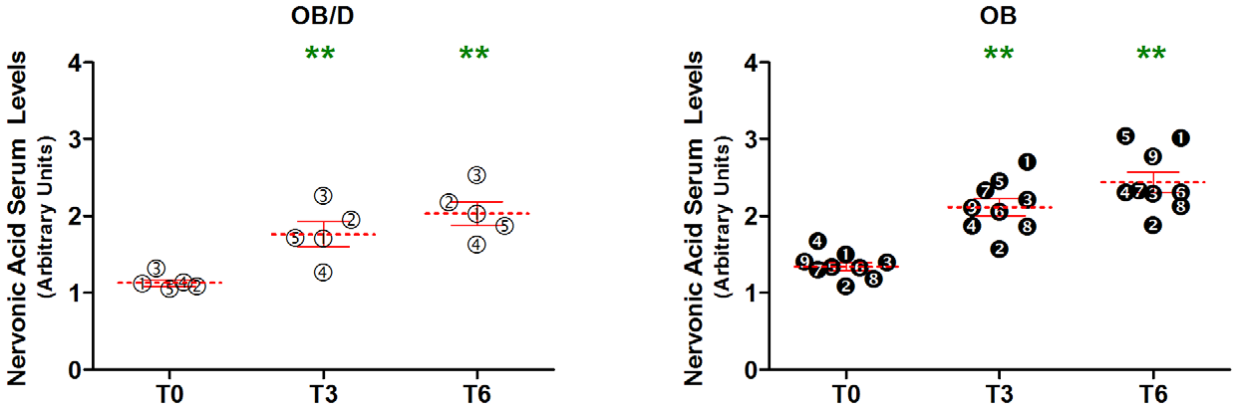
Increases in nervonic acid following RYGB. A significant increase in serum abundance of nervonic acid occurred following RYGB in both OB and OB/D subjects, as assessed using a mixed-effects ANOVA with group:time interaction (** *p*<0.01 versus T0). Overall, nervonic acid levels were significantly different at each time point between OB and OB/D subgroups (p<0.01, see **TABLE 2**). No group:time interaction was identified. Mean metabolite abundance±SEM is indicated in red.

We then integrated clinical and metabolite data from all three time points in the 12 non-insulin treated subjects with the goal of determining whether any of the aforementioned metabolites could be used as ‘candidate’ markers that reflect the clinical adaptations seen following RYGB. Independent Spearman correlations were performed for each of the 8 aforementioned metabolites versus BMI and HOMA-IR. Of the 8 metabolites examined, we identified a significant negative correlation between nervonic acid and HOMA-IR (p = 0.001, R = −0.55), i.e. HOMA-IR decreased (**FIGURE 2**) as serum levels of nervonic acid increased (**FIGURE 5**). This relationship was similarly observed when considering only the 9 OB subjects independently (p = 0.008, R = −0.51). Furthermore, adjusting for BMI did not affect the significance of the correlation between nervonic acid and HOMA-IR. No relationships were identified when correlating the delta values for changes in BMI and HOMA-IR with these 8 metabolites (data not shown).

## Discussion

Gastric bypass surgery results in significant metabolic changes associated with weight loss, improved insulin sensitivity and glucose homeostasis, and a reduction in co-morbidities such as diabetes and coronary heart disease. A retrospective study in approximately 10,000 subjects who had undergone gastric bypass surgery revealed that mortality related to diabetes decreased by over 90%, leading to this medical procedure being proposed as a means to treat diabetes, even in subjects who are obese rather than morbidly obese [3]. As such, there is significant interest in unraveling the molecular adaptations underlying the improved insulin sensitivity seen post RYGB. We performed a global metabolite profiling analysis in morbidly obese female subjects before and after RYGB in order to generate new knowledge regarding the physiological and metabolic changes arising with this procedure.

All 14 patients experienced an improvement in the clinical parameters measured. Decreases in BMI, body weight, fat mass, fat free mass, triglycerides, insulin resistance (HOMA-IR), and leptin were observed following RYGB. Subjects also experienced a decrease in resting energy expenditure. Furthermore, while total caloric intake decreased dramatically post surgery, the relative proportion of protein, lipid, and carbohydrate consumed in the diet remained stable. The OB/D subjects had additional physiological improvements post surgery related to their diabetes status. OB/D subjects experienced a drop in HbA1C levels at 3 months post RYGB. Three of the 5 OB/D subjects demonstrated a resolution of diabetes, as suggested by the normalization of glucose levels and the fact they no longer needed anti-diabetic and dyslipidemia treatments after 3 months. The remaining 2 OB/D subjects demonstrated improvements in their diabetic status, but insulin therapy was still required. Interestingly, these two subjects both had diabetes for 11 years prior to surgery and higher pre-surgery HbA1c levels in contrast with the other three subjects, who had diabetes for 5 years prior to surgery and lower pre-surgery HbA1c levels. This suggests the RYGB surgery may be more efficacious for resolving diabetes if performed soon after diagnosis in subjects with low HbA1C levels.

In the present study we have identified 83 serum metabolites whose abundance changed significantly following RYGB; where the majority of these metabolites corresponded to lipids and amino acids. The use of a stringent Bonferroni adjustment to correct for multiple testing highlighted the large number of metabolites that decreased immediately post surgery and then did not change between T3 and T6 (**FIGURE 1**). To the best of our knowledge, previous lipid analyses before and after gastric bypass surgery have only focused on lipoproteins and lipid classes, but not individual lipid species. The improvements in dyslipidemia associated with gastric bypass were previously demonstrated by increases in HDL-cholesterol and reductions in total cholesterol, LDL-cholesterol, and triglycerides [6], [27]. More recently, Schwab *et al*. performed an analysis of the lipid-fraction of the serum metabolome in subjects before and after diet-induced weight loss. Although diet restriction is a considerably less dramatic weight loss situation than RYGB, the authors found that diet-induced weight loss led to decreases in triglycerides and phospholipids, but no changes to lysophosphatidylcholine (LPC) and sphingolipid species [13]. The lack of changes in LPC and sphingolipids was surprising when considering previous animal studies have shown that LPC species and sphingolipids are increased with obesity and in situations marked by impaired insulin sensitivity [14], [15], [28]; however, it is plausible that less drastic weight-loss paradigms such as exercise and diet restriction do not affect insulin sensitivity sufficiently to detect changes in LPC and sphingolipids.

While many lipid species were found to increase in subjects post RYGB, several lipids were of particular interest, namely nervonic acid and various sphingosine species (3-O-methylsphingosine, threo-sphingosine, erythro-sphingosine, and 5-O-methylsphingosine). In light of recent evidence regarding the role of intramyocellular lipids (IMCL) and insulin resistance, it is interesting to speculate that plasma increases in the aforementioned lipids reflect their mobilization from non-adipose tissues such as the muscle. Elevated IMCL deposition has been observed in obese individuals, obese subjects with type 2 diabetes and insulin resistant subjects [29], [30]. One year after elective gastric bypass surgery, Gray et al. found a ∼30% decrease in IMCL and hypothesized that decreases in muscle lipid content may contribute to improved insulin action [29]. More specifically, intramyocellular sphingolipids may cause insulin resistance by disrupting insulin signaling pathways in the muscle [31]. Previous reports have revealed that the level of muscle ceramide enriched with nervonic acid is negatively correlated with insulin sensitivity, but the authors did not examine plasma lipid levels. Recently, Haus and colleagues have found that serum ceramide enriched with nervonic acid is inversely correlated with insulin sensitivity [30]; however, the authors did not determine free levels of circulating nervonic acid. In light of our findings demonstrating increases in total serum nervonic acid post RYGB and its negative correlation with insulin resistance, it would be interesting to simultaneously generate metabolomic datasets in both the muscle and plasma before and after RYGB in order to determine whether the release of IMCL is responsible for the increased serum lipid levels we observed. Additionally, it will be interesting to determine whether decreases in IMCL correlate with the loss of fat free mass, also observed post gastric bypass surgery [32], [33]. This would provide further insight into the flux of lipids and reveal whether IMCL are mobilized into serum following RYGB.

Our work also revealed that branched chain amino acids (BCAA) serum levels decreased following RYGB. The decrease in valine was paralleled by an increase in serum β-aminoisobutyric acid, a product of valine catabolism. Our results are in line with a previous report indicating that plasma BCAA decrease by approximately 35% following gastric bypass surgery [34]. The subjects in this independent study showed a significant decrease in body weight and plasma glucose levels, but no improvement in insulin sensitivity; however, this may be a result of the increased inter-individual variability associated with the use of a mixed-sex cohort. The authors also demonstrated an increase in both subcutaneous and visceral adipose tissue mitochondrial BCAA aminotransferase (BCATm) protein levels, a key enzyme involved in BCAA catabolism. As such, the authors proposed that lower plasma BCAA levels post gastric bypass surgery reflect an increase in adipose tissue BCAA catabolism. The same authors demonstrated elevated plasma BCAA levels in a BCATm −/− mouse model [35]; however, differences between mice and humans can be noted in these studies. Specifically, elevated BCAA levels were associated with improved glucose and insulin tolerance in BCATm−/− mice, while decreases in BCAA levels are associated with improved glucose and insulin tolerance in humans following RYGB. The results in humans were recently reinforced by two independent studies and demonstrated that BCAA plasma levels are higher in obese subjects versus their non-obese subjects [16], [36]. Furthermore, the expression of genes associated with the BCAA catabolic pathway were significantly decreased in subcutaneous adipose tissue of obese twins, and this was additionally associated with insulin resistance [36]. This suggests that high levels of circulating BCAA are associated with an insulin resistant state, as recently discussed by Newgard et al [16]. Thus it can be hypothesized that increases in BCAA catabolism result in lower circulating BCAA levels which are associated with improved insulin sensitivity in humans. Our results agree with this hypothesis, as all three BCAA decrease post RYGB where insulin sensitivity improves.

Because this is the first study examining metabolic changes following RYGB, we have chosen to briefly discuss some metabolites that were significantly different at an unadjusted 0.05 p-level to highlight potentially promising metabolites for further study. These metabolites provide intriguing and novel information illustrating the many physiological adaptations arising post RYGB; although their relationship with RYGB, morbid obesity, and diabetes has not been extensively studied. For example, RYGB was previously shown to improve hypothyroidism [37]. We have identified a significant increase in thyroxine (T4), the major form of thyroid hormone found in blood, post surgery that may underlie the improvement in thyroid function and signaling seen in subjects after RYGB. The abundance of protein-bound uremic retention solutes (p-cresol sulfate, indole-3-acetic acid, 3-indoxylsulfuric acid, and 3-hydroxyindole) was increased post RYGB and, several of the precursor metabolites were decreased after bypass surgery, such as tyrosine, phenylalanine, and tryptophan [38]. Serum increases in these metabolites are typically associated with renal failure; however, the increased abundance of these metabolites may be a consequence of modifications to the gut environment [39]. Indeed, p-cresol and indole are metabolites generated by the intestinal microbiota, suggesting that RYGB may lead to alterations in the gut microbial composition. The serum abundance for a number of phytochemicals was also altered following gastric bypass surgery. More specifically, beta-sitosterol and campesterol were decreased, and lycopene and beta-carotene were increased. Phytochemicals are bioactive, non-nutritional molecules derived from plant sources and whose consumption is generally associated with a reduced risk for various diseases, such as cardiovascular disease and cancer [40], [41]. Although controversial, plasma increases in plant sterols (e.g. campesterol and beta-sitosterol) have been associated with an increased risk for coronary heart disease [42]; thus our data may suggest RYGB reduces the abundance of these risk factors. Phytochemicals can be absorbed to a certain extent all along the gastrointestinal tract; however, they are predominantly absorbed in the duodenum and jejunum [40]. Therefore it is possible that the changes observed in serum phytochemical abundance is a consequence of the physical restructuring of the gastrointestinal tract during RYGB surgery that involves bypassing much of the proximal small intestine. Although the mechanisms underlying changes in the serum abundance of the aforementioned metabolites is unclear at present, our study has yielded novel and intriguing avenues of research that will ultimately provide a deeper understanding of the numerous physiological and metabolic consequences of gastric bypass surgery.

We further studied whether changes in metabolite abundance were associated with diabetes by separating our cohort into obese and obese/diabetic subjects. We felt this analysis was justified and valid for several reasons. Firstly, subgroup analysis between OB and OB/D subjects was only performed when statistical significance was achieved according to Akaike information criterion. Secondly, metabolite profiling revealed that glucose was greater in OB/D subjects compared to OB subjects at all three time points (p<0.05). Thirdly, the metabolite that differed the most between OB and OB/D subjects was 1,5-anhydrosorbitol (p<0.001), a biomarker currently used for assessing glycemic control [24]. This metabolite was more abundant in OB/D serum. Furthermore, blood levels of 1,5-anhydrosorbitol are inversely related to glucose, which our data also confirms. Finally, OB/D subjects showed a marked improvement in their diabetic status. Three of the five OB/D subjects demonstrated a resolution of diabetes and no longer required treatments related to diabetes or dyslipidemia. Thus, any differences between OB and OB/D that may be related to pre-surgery diabetes treatment would have less importance post surgery. Nevertheless, to avoid this potential confounder, we have only focused on metabolites that are systematically identified at all three time points to differentiate OB and OB/D subjects. Eight metabolites were found to both differentiate OB from OB/D subjects consistently at all time points and change over time (T0 vs. other two time points T3, T6): lycopene, asparagine, lysophosphatidylcholine (C18:2), p-Cresol sulfate, lactate, nervonic acid (C24:cis[15]1), glucose, and mannose. We integrated data for the 8 aforementioned metabolites with the clinical data available for our subjects regarding BMI and HOMA-IR. Nervonic acid was found to be negatively correlated with HOMA-IR, which is of particular interest considering that this fatty acid has previously been positioned as having preventive effects on obesity-related metabolic parameters [43]. While little else is known regarding its functional role in obesity, it will be interesting to determine whether nervonic acid induces or is simply a consequence of the metabolic improvements seen post RYGB.

In summary, applying global metabolite profiling to the study of subjects before and after RYGB has provided considerable new information regarding the numerous alterations to serum metabolite levels arising with this surgical procedure. While we have identified metabolites not previously associated with the physiological and metabolic alterations arising with gastric bypass surgery, it is imperative that the next step taken in unraveling the physiological improvements following RYGB uses a systems biology approach to study metabolite fluxes between serum, muscle, liver, and adipose tissue. The additional knowledge regarding the communication between tissues and body fluids will permit a better understanding of whether metabolite changes are responsible for, or an indirect effect of, clinical improvements.
